# NEK8, a NIMA-family protein kinase at the core of the ciliary INV complex

**DOI:** 10.1186/s12964-025-02143-w

**Published:** 2025-04-07

**Authors:** Joan Roig

**Affiliations:** Department of Cells and Tissues, Cell Cycle and Signaling Research Group, Molecular Biology Institute of Barcelona (IBMB-CSIC), Baldiri I Reixac 10-12, Barcelona, 08028 Spain

**Keywords:** Protein kinase, NIMA family, NEK, INV compartment, Cilia

## Abstract

Here we describe the current knowledge about the ciliary kinase NEK8, highlighting what we know and what we don’t know about its regulation, substrates and potential functions. We also review the literature about the pathological consequences of different *NEK8* variants in patients of nephronophthisis, renal-hepatic-pancreatic dysplasia and autosomal dominant polycystic kidney disease, three different types of ciliopathies. NEK8 belongs to the NIMA family of serine/threonine protein kinases. Like its closest relative, NEK9, it contains a protein kinase and an RCC1 domain, but lacks the C-terminal region that is key for NEK9’s regulation as a G2/M kinase. Importantly, NEK8 localizes to cilia as part of a multimeric protein complex that assembles in a fibrillar fashion at the proximal half of this signaling organelle, defining what is known as the INV compartment. NEK8 and its INV compartment partners inversin, ANKS6 and NPHP3 are necessary for left–right determination and the correct development of different organs such as the kidney, the heart and the liver. But the kinase substrates, regulatory mechanism and activating cues and thus the molecular basis of NEK8 important physiological roles remain elusive. We present the current findings regarding NEK8 and also highlight what we miss in order to progress towards the understanding of the kinase and the function of the INV complex at the cilia.

Primary cilia are hair-like organelles that protrude from the cell body and are present in most animal cells. 1–20 μm in length, they are formed by cell membrane surrounding an internal microtubule structure, the axoneme, organized by the centrosomal mother centriole, called in this context the basal body. Cilia have a very specific lipid and protein composition that allows them to act as sensory organelles, receiving information from other cells and the environment and integrating it in order to change cell physiology. Responding to mechanical and chemical cues, such as liquid flow and different signaling molecules, they are key to tissue development, maintenance and function [1, 2]. Accordingly, pathogenic variants of ciliary proteins result in ciliopathies, an heterogenous group of diseases that present with a wide range of clinical phenotypes including cystic-fibrotic kidneys and liver, skeletal deformities, retinal degeneration, mental retardation, laterality defects and congenital heart disease [3–5].

Nephronophthisis (NPHP) are a group of autosomal recessive ciliopathies characterized by progressive damage to the nephrons, the kidneys filtering units [4, 6–8]. This damage leads to chronic kidney disease and eventually end-stage renal disease. Nephronophthisis can be classified into subtypes based on the age of onset. Thus, the most common form, juvenile nephronophthisis, is characterized by the occurrence of end-stage renal disease in adolescence or young adulthood, with symptoms appearing as early as 6 years of age. Infantile nephronophthisis is very rare and presents in early infancy with severe kidney dysfunction, often leading to kidney failure prior to 4 years of age. Adolescent nephronophthisis manifests later in adulthood with a slower progression of kidney damage and a mean age of onset of end-stage renal disease of 19 years. Nephronophthisis tipically presents with fibrotic kidneys with small cysts located in the corticomedullary region (although the degree of fibrosis and the number/size of cysts can vary significantly across nephronophthisis subtypes, disease stages and the specific causing genetic mutations). This contrasts with polycystic kidney disease (PKD), which is characterized by enlarged kidneys containing large cysts distributed across multiple segments of the nephron (note that in infantile forms of nephronophthisis cysts can also cause kidney enlargement). The most frequent cause for kidney failure in the first 3 decades of life, nephronophthisis can be isolated, or depending on the causing variants and possibly the presence of different modifier genes, it can present in syndromic forms that involve different extrarenal manifestations such as liver fibrosis, retinal degeneration (Senior-Løken syndrome), cerebellar vermis hypoplasia (Joubert syndrome), cardiac and skeletal anomalies, ectodermal dysplasia, heterotaxy or *situs inversus*, and/or brain malformations and mental retardation. In some cases, it partially overlaps with other syndromic pathologies such as Meckel-Gruber syndrome.

More than 20 *NPHP* genes have been identified to date, mostly coding for proteins that reside at the cilium or the centrosome/basal body. The pathologies caused by variants of these genes are accordingly thought to be the result of ciliary malfunction, possibly resulting in abnormal cell divisions and/or the failure to establish an adequate tissue structure. In the kidney this would disrupt epithelial tubular structure and give rise to nephritis and the formation of cysts. Other tissues could be affected similarly, i.e. in view of the observed phenotypes NPHP mutations could conceivably affect the functions of ciliated cells in the embryonic node (in charge of generating a leftward fluid flow that is crucial for establishing left–right asymmetry during development), cholangiocytes, the ciliated epithelial cells lining bile ducts in the liver, or the cilia of retinal photoreceptors. And, as mature cardiomyocytes lack cilia, cardiac phenotypes could be the indirect result of a requirement for NPHP proteins in embryonic cardiac cilia or, alternatively, a secondary effect of heterotaxy as a result of abnormal left–right determination.

NEK8, the object of this review, is a serine-threonine protein kinase of the NIMA-family that localizes at the cilia. Different variants of the *NEK8* gene (accordingly also named as *NPHP9*) have been found to associate with infantile nephronophthisis and other developmental pathologies, indicating that NEK8 has important and necessary functions during the development of the kidneys and other organs. What these functions are is unclear. This work summarizes current knowledge of NEK8 to help elucidate them and the pathologies associated with deleterious *NEK8* variants in animal models and human patients. Importantly, NEK8 is a protein kinase and understanding its functions will likely mean understanding how it controls different substate proteins through phosphorylation. Thus, we will describe what is known about its structure, kinase activity, regulation and substrates and also relate it to its closest evolutionary relatives in the NIMA family.

## The NIMA family of protein kinases

Protein kinases can be grouped into several families based on the evolutive relationships of their catalytic domains. Additional regulatory domains or regions usually contribute to confer a distinct set of characteristics to each specific kinase, providing for example regulation, subcellular localization cues or the ability to oligomerize or to interact with other proteins (consequently, mutations in the regulatory domains can have similar or even more detrimental consequences for the function of a protein kinase than these directly modifying its enzymatic domain).

The human genome codes for two protein kinases, NEK8 and NEK9, that contain an RCC1-like regulatory domain, a region with homology to the Regulator of Chromosome Condensation 1 (RCC1) Ran GTPase exchange factor [9], that functionally acts as a protein interaction module. They are both closely related and belong to the NIMA family, a group of serine/threonine kinases conserved across most eukaryotes that has important roles regulating the cell cycle and the microtubule cytoskeleton [10–12]. The NIMA kinases are named after its founding member, *Aspergillus nidulans* NIMA, a protein encoded by the *nimA* gene that was identified in a genetic screen as the first of a group of genes that when mutated blocked cells in interphase, hence Never In Mitosis A [13, 14]. *Aspergillus* NIMA is a key regulator of the cell cycle and specifically G2 and mitosis, controlling chromosome condensation, nuclear envelope breakdown and spindle organization (reviewed in [10]). It is clear that members of the NIMA family share similar functions in different organisms including humans, but also current evidence suggests that the NIMAs (also called NEKs or NKLs, depending on the organism) have acquired important roles not related to cell cycle control, for example in the DNA-damage response or the control of cilia function.

The number of NIMA-family members varies significantly across eukaryotes and it is thought to be related to the complexity of the regulatory needs of the centrosome/basal body and cilia/flagella apparatus in different organisms. Thus, while non-ciliated fungi have a single NIMA kinase, the multiciliated *Tetrahymena* has almost forty and *Giardia* more than a hundred [15, 16]. Regarding well studied model organisms, *Drosophila* has two and *C. elegans* has four.

**Fig. 1:**
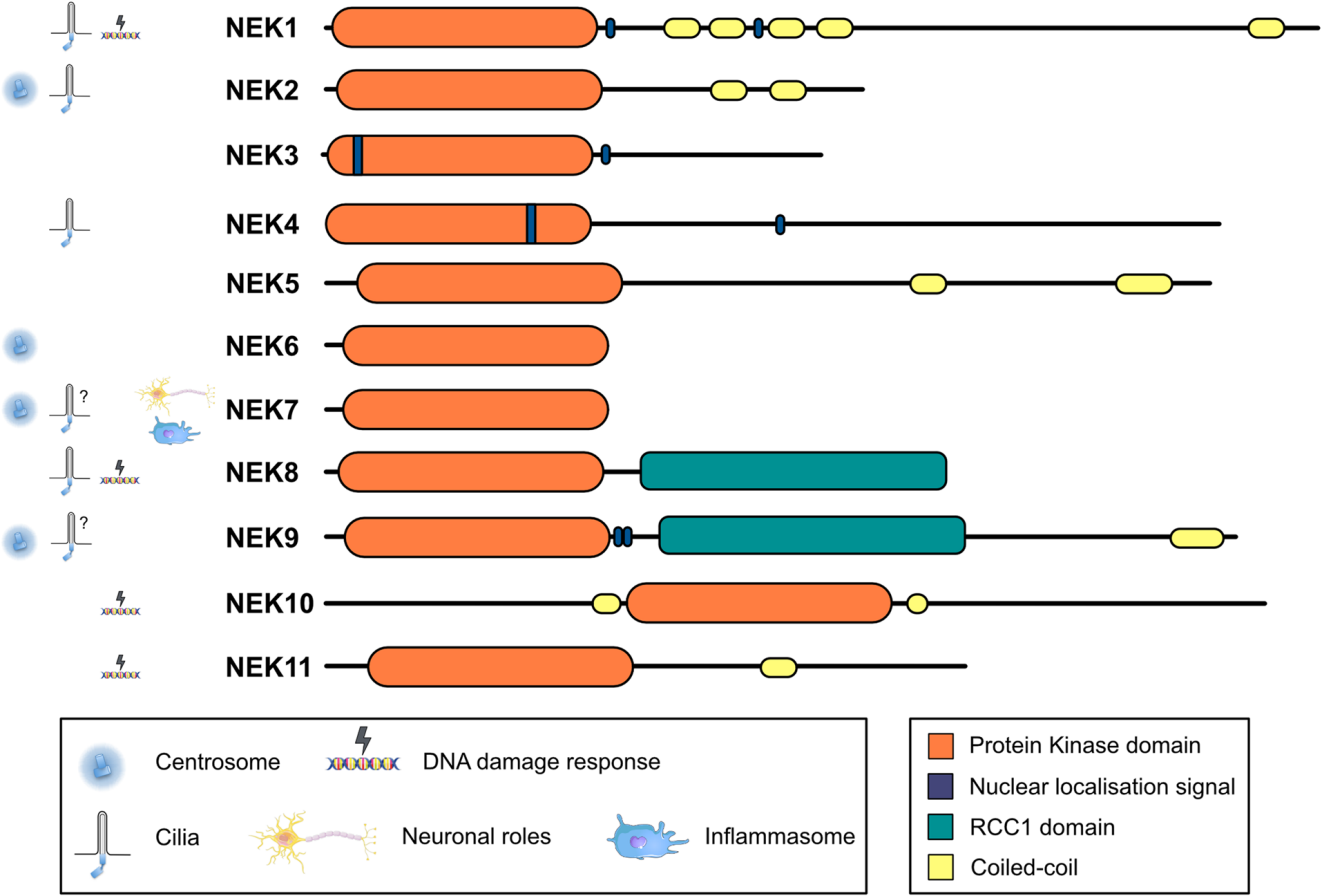
The NIMA family of protein kinases in humans. The different proteins and their known domains are shown. Suggested functions of the NEKs are indicated. See [12] for references. Neuron, macrophage and DNA images from Servier Medical Art templates (licensed under CC BY 4.0; https://smart.servier.com)

Humans have 11 NIMA-family kinases, called NEK1 to NEK11 (Fig. 1). They contain an evolutionarily related protein kinase domain usually followed by a regulatory region with different domains or motifs. The best understood of them, NEK2, NEK9, and the highly similar NEK6 and NEK7, are like *Aspergillus* NIMA involved in the regulation of different aspects of the G2 and M phases of the cell cycle, acting downstream of cyclin-dependent kinases and the Polo-like kinase PLK1. Cell cycle-related functions of the NEKs have been reviewed elsewhere [11, 12] and will not be covered here.

### Ciliary functions of the mammalian NIMA kinases

Importantly for this review, several NIMA family kinases have been shown to have functions related to the primary cilium. The relationship between NEK8 and cilia will be described at length below. We will here briefly outline what is known about other NEKs in relation to the cilium (also reviewed in [17]).

The original connection between cilia and the NIMA family was established in 2000, when a mutation that caused pleiotropic effects including PKD (characterized by the grow of cysts in the kidneys eventually leading to organ failure) in two mouse strains named kat and kat(2 J) was mapped to the *Nek1* gene [18]. Bi-allelic variants in *NEK1* were subsequently proposed to cause a human ciliopathy, short-rib polydactyly syndrome type Majewski [19] and variants of *NEK1* have since also been involved in the onset of amyotrophic lateral sclerosis [20]. NEK1 was shown by the groups of Lynn Quarmby and Benny Motro to localize to the centrosome in cycling cells and to the basal body in ciliated cells, and to be necessary for normal ciliogenesis. It has thus been proposed to coordinate the cell division cycle with the centrosome/cilia cycle [21–23]. Regarding other NEKs, the Motro group has also shown that NEK7-deficient cells have abnormal cilia numbers, thus suggesting that this kinase is like NEK1 somehow involved in the control of the formation and maintenance of primary cilia [24]. NEK7 though seems to be involved in the regulation of the centriole cycle and its misfunction has been related to the apparition to abnormal centriole numbers [25]. Thus, as other kinases with similar functions at the centriole, it may be only indirectly linked to the control of cilia. This may also be the case for NEK9, the upstream activator of NEK7 (and close relative of NEK8) as homozygous stop-gain mutations in the *NEK9* gene result in a lethal skeletal dysplasia possibly characterized by ciliary defects [26]. To our knowledge, though, neither NEK7 nor NEK9 have convincingly been shown to localize to cilia or basal bodies in quiescent cells. Puzzlingly, NEK9 may be directly involved in ciliogenesis via a kinase-independent role regulating autophagy [27].

The centrosomal kinase NEK2 does localize to the basal body in ciliated cells and is involved in cilia resorption, possibly through the regulation of different proteins such as Kif24, a microtubule depolymerizing kinesin, and the tubulin deacetylase HDAC6 [28–30]. NEK4 has also been shown to localize to basal bodies and rootlets and regulate primary cilium stability through unknown mechanisms [31].

Finally, although not being a ciliary kinase per se, NEK10 has been shown to regulate protein composition, length and motion of motile cilia and to be necessary for normal mucociliary clearance in the airways [32].

## NEK8, a ciliary NIMA-family kinase

**Fig. 2:**
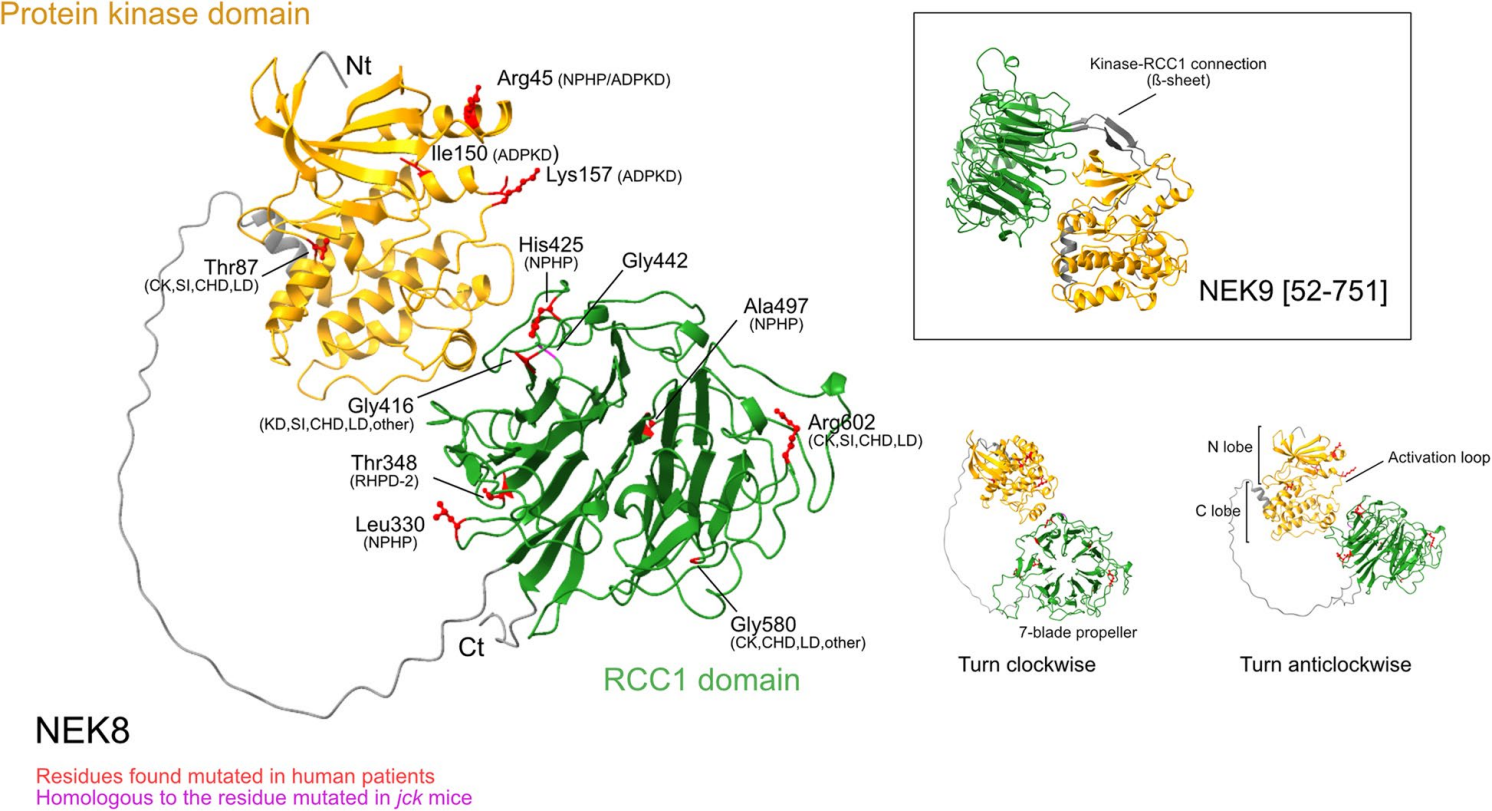
NEK8 predicted structure. Some of the residues with pathogenic variants in human patients are highlighted in red, clinical diagnosis is indicated (*NPHP*, nephronophthisis; *RHPD*, renal-hepatic-pancreatic dysplasia; *ADPKD*, autosomal dominant polycystic kidney disease; *CHD*, congenital heart disease; *CK*, Cystic kidneys; *KD*, kidney defects, possibly no cysts; *LD*, liver defects; *SI*, *situs inversus*). Gly442, the homologous residue to the mouse Gly448 mutated in the *jck* animals is shown in magenta. See also Table 1. The predicted structure of NEK9 residues 52–751 is also shown to illustrate the differences in connection between domains in each kinase. Structures predicted using Alphafold [33] and modified in ChimeraX [37]

NEK8 (also known as NPHP9) is a 692 residues protein of ~ 75 kDa that in humans is encoded by chromosome 17 (uniprot Q86SG6). As expected from its sequence NEK8 is predicted by Alphafold [33] to have a structure consisting of an N-terminal bilobal domain characteristic of protein kinases [34] plus a C-terminal RCC1-related domain with a seven blade β-propeller structure [9] (Fig. 2). These two well-structured domains are linked by a region that is most probably disorganized and flexible. No clear interaction surfaces are predicted in between the kinase and the RCC1 domain, suggesting that the two domains are positioned independently, at least in absence of any NEK8 interactors. This is in contrast with NEK9, which is predicted to have its kinase and RCC1 domains connected by a region structured as a beta sheet formed by two antiparallel beta strands (Fig. 2, inset). This connection may keep the two domains of NEK9 tightly connected and in fact they have been shown to interact in vivo. It may also account for the inhibitory effect that the RCC1 domain has on the protein kinase domain of NEK9, something that is not observed in NEK8 [35, 36]. See below for a discussion of the regulation of NEK8.

**Table 1:** NEK8 mutations. Published NEK8 mutations, including experimental mutations and pathological mutations in both animal models and human patients. *NPHP*, nephronophthisis; *RHPD*, renal-hepatic-pancreatic dysplasia; *ADPKD*, autosomal dominant polycystic kidney disease; *CHD*, congenital heart disease; *SI*, *situs inversus*

1- Experimental kinase domain mutations				
**Mutation**	**Protein region**	**Effects on protein**	**Reference**	
Lys33Met	Kinase domain (ATP interaction)	Abrogates activity As wt, rescues both ANKS6 and NPHP3 INV compartment localization to WT-like densities in NEK8-KO cells	Liu et al. 2002 [38] Trapp et al. 2008 [39] Benett et al. 2020 [40] Czarnecki et al. 2015 [41]	
Lys33Met, Asp128Ala	Kinase domain (ATP interaction, proton acceptor)	Abrogates activity, affects centrosomal and ciliary staining	Zalli et al. 2012 [36]	
Thr162A	Kinase domain (activation loop)	Abrogates activity, affects centrosomal and ciliary staining	Zalli et al. 2012 [36]	
2- Pathological animal mutations				
**Mutation**	**Protein region**	**Effects on protein**	**Pathological consequences (homozygous)**	**Reference**
Mouse Gly448Val (human Gly442Val); *jck* mutation	RCC1 domain	Changes subcellular localization: loss of centrosomal and ciliary localization; reported both to affect and not affect kinase activity. Does not affect interaction with NPHP3	Juvenile cystic kidneys DNA damage elevated in kidneys	Liu et al. 2002 [38] Also data from: Zalli et al. 2012 [36] Choi et al. 2013 [42] Bennet et al. 2020 [40]
*Nek8* ^−/−^ mice	-	-	Randomized left–right asymmetry, cardiac abnormalities and kidney cysts; die shortly after birth	Manning et al. 2013 [43]
Mouse Ile124Thr (human Ile124Thr); Roc mutation	Kinase domain (close to proton acceptor)	Kinase deficient; does not affect ciliary localization	Laterality defects, cardiopulmonary malformations, cystic kidneys	Czarnecki et al. 2015 [41]
Rat Arg650Cys (human Arg644Cys); Lewis polycystic kidney mutation	RCC1 domain	Altered ciliary localization; longer cilia	NPHP	McCooke et al. 2012 [44]
3- Examples of pathological human mutations				
See also https://www.ncbi.nlm.nih.gov/clinvar/				
**Mutation**	**Protein region**	**Effects on protein**	**Pathological consequences (for clinical details see reference)**	**Reference**
p.Leu330Phe	RCC1	Decreased ciliary (but not centrosomal) localization; no effect on kinase activity	NPHP; not clear whether renal cysts are present; retinitis pigmentosa, blindness at age 24 Not clear if causative, patient has mutation heterozygously and carries homozygous mutation in *NPHP5*	Otto et al. 2008 [45] Also data from: Zalli et al. 2012 [36]
p.His425Tyr	RCC1	Absent from to cilia and decreased localization to the centrosome; diminished ANKS6 at the cilia; reported both to affect and not affect kinase activity; interferes with interaction between NEK8 and TAZ and abrogates NEK8-dependent TAZ activity	NPHP Homozygous	Otto et al. 2008 [45] Also data from: Zalli et al. 2012 [36] Habbig et al. 2012 [46] Choi et al. 2013 [42] Grampa et al. 2016 [47]
p.Ala497Pro	RCC1	Slightly decreased ciliary (but not centrosomal) localization; no effect on kinase activity	NPHP; not clear whether renal cysts are present Heterozygous ?	Otto et al. 2008 [45] Also data from: Zalli et al. 2012 [36]
p.Arg599Ter	RCC1	No protein (nonsense-mediated mRNA decay) Patient cells showed decreased PKD1 and PKD2 expression, c-MYC upregulation, downregulation of the TAZ/Hippo signaling pathway	Non viable or pregnancy terminated Early embryonic phenotype with cystic- dysplastic kidney, liver and pancreas; CHD; heterotaxy; other developmental abnormalities Homozygous	Frank et al. 2013 [48]
p.Thr348Met	RCC1	Not determined	Perinatal or early death RHPD; Cystic kidneys; liver abnormalities; CHD Compound heterozygous	Rajagopalan et al. 2015 [49]
p.Ter693LeufsTer86	C-terminal end			
p.Thr87Ala	Kinase, ATP binding region	Patient fibroblasts (T87A, R602W) showed absence of NEK8 in the cilia, but presence in the Golgi apparatus; slightly reduced percentage of ciliated cells; shorter cilia T87A alone: partial localization to cilia; significant decrease in ANKS6 binding R602W alone: loss of cilia location; diminished ANKS6 at the cilia; diminished cilia number	Early death Cystic kidneys, SI, CHD, liver defects Compound heterozygous	Grampa et al. 2016 [47]
p.Arg602Trp	RCC1			
p.Gly580Ser	RCC1	Loss of cilia localization; diminished ciliation; diminished ANKS6 at the cilia	Early death Cystic kidney; CHD, liver defects; other developmental abnormalities; (SI in fetus sibling) Homozygous	Grampa et al. 2016 [47]
p.Val163Ala206del	Kinase (region includes part of the activation loop)	Not determined	Pregnancy terminated Renal defects (no cysts?); SI; CHD; liver and pancreas abnormalities; other developmental abnormalities Compound heterozygous	Grampa et al. 2016 [47]
p.Gly416Ser	RCC1	Not determined		
Splicing defect (c.47 + 1 G > A, intron 1)	-	-	Pregnancy terminated Cystic kidneys; SI; pancreas cysts; other developmental abnormalities Homozygous	Grampa et al. 2016 [47]
p.Arg127Ter	Kinase	Patient fibroblasts show absence of NEK8 in the cilia, possibly the result of loss of protein expression Ciliogenesis not affected; loss of ANKS6 immunostaining at the cilia	Pregnancy terminated Cystic kidneys; SI; liver and pancreas abnormalities; other developmental abnormalities Compound heterozygous	Grampa et al. 2016 [47]
p.Arg462Ter	RCC1			
p.Trp467Ter	RCC1	Not determined	Fetal death Cystic kidneys; other developmental abnormalities Presumed homozygous	Al-Hamed et al. 2016 [50]
p.Arg45Trp	Kinase	Reduced kinase activity (?) Expression and localization at cilia normal; no obvious effects on ciliogenesis; results in decreased polycystin-2 but normal ANKS6 localization in cilia; does not rescue DNA damage in -/- cells	Infantile NPHP/ADPKD; chronic anemia; No other extra-renal features Heterozygous; de novo; autosomal dominant mutation; recurrent	Claus et al. 2023 [51] Mehawej et al. 2023 [52] Elhassan et al. 2024 [53]
p.Ile150Met	Kinase, activation loop	Expression and localization at cilia normal; no obvious effects on ciliogenesis; results in normal polycystin-2 and ANKS6 localization in cilia	ADPKD; mild phenotype Heterozygous; autosomal dominant mutation	Claus et al. 2023 [51]
p.Lys157Gln	Kinase, activation loop	Expression and localization at cilia normal; no obvious effects on ciliogenesis; results in decreased polycystin-2 but normal ANKS6 localization in cilia; patient fibroblasts show DNA damage	ADPKD; bilateral PKD; no other extra-renal features Heterozygous; autosomal dominant mutation	Claus et al. 2023 [51]
p.Asn69Asp	Kinase	Not determined	ADPKD; absence of liver and pancreatic cysts Heterozygous; autosomal dominant mutation	Elhassan et al. 2024 [53]
p.Arg140Leu	Kinase	Not determined	ADPKD; absence of liver and pancreatic cysts Heterozygous; autosomal dominant mutation	Elhassan et al. 2024 [53]

NEK8 was originally described in 2002 by David Beier and colleagues as the product of a gene that when mutated resulted in PKD in mice [38]. Thus, from the very beginning research on the kinase has been linked to disease. It is important to note that almost simultaneously a kinase that was named “Nek8” but also “Nercc1” was described by others and us [35, 54]. This protein was later renamed as NEK9, and as mentioned above it is the closest relative of the bona fide NEK8 (see Figs. 1 and 2).

Human NEK8 was predicted in the original work describing mouse NEK8 [38] as well as in the analysis of the human kinome [10, 55]. It was subsequently cloned as a result of a search for novel protein kinases [56].

The Beier group found that the mutation causing the pathology in *jck* mice, a model of autosomal recessive PKD, was a change from glycine to valine (Gly448Val) in the RCC1 domain of NEK8 [38], see Fig. 2. Notably, after 20 years of this discovery the exact functional consequence of this mutation remains obscure. Beier et al. suggested that it affected NEK8 subcellular localization (the kinase was then not yet known to localize at the cilium), something that is supported by the work of other authors, that have shown that this (or the homologous G442V in humans) and other functionally related mutants of NEK8 are improperly localized [36, 39]. Other reports, though, find the localization of the *jck* mutant similar to that of wild type [40]. Contradictory results have also been published about the effect of the *jck* mutation on the protein kinase activity of NEK8. While some researchers have shown that the mutation does not significatively affect the activity of the kinase [36, 41], others have reported that the *jck* mutant functionally behaves as a kinase-deficient form of the enzyme [42]. Clearly the molecular consequences of the *jck* mutations needs to be explored further.

The causal correlation between an impaired NEK8 function and the appearance of kidney cysts has been confirmed in zebrafish [38]. Interfering with the expression of the ortholog of NEK8 in this system was observed to result in the appearance of pronephric cysts during early development, something subsequently confirmed by other groups, that also observed abnormal cardiac looping, anticipating yet another aspect of the NEK8-related pathologies [38, 47, 57]. The phenotype observed in both NEK8-compromised mice and zebrafish seems to be the result of a failure to maintain normal kidney epithelia structure. Thus, in *jck/jck* mice, cell adhesion to the basal membrane in tubular epithelia is disrupted [38] and altered cell–cell junctions associated to abnormal expression of E-cadherin and desmosomal proteins has been observed in cystic kidney organ cultures derived from the same mice [58]. Similarly, in zebrafish, pronephric duct cells detached from the basement membrane and were observed in the duct lumens. Overexpression of the *jck* mutant or a kinase-deficient form of the enzyme (NEK8 Lys33Met) in different types of cells resulted in changes in cell morphology, further supporting the idea that abnormal NEK8 signaling can indeed induce cellular changes that result in structural aberrations at the tissue level and the formation of cysts [38].

Besides mice and zebrafish, the importance of NEK8 for normal kidney development has also been corroborated in humans (see “NEK8-associated pathologies in humans” below) and rats [44]. Further research has shown that the kinase is also important for the development of other organs besides the kidney, i.e. the heart and liver, as well as for the establishment of left–right laterality. In fact NEK8 is expressed in most tissues in mice [38], and humans (e.g. https://www.proteinatlas.org).

The original description of NEK8 reported it to be localized to the apical region of epithelial cells, but it is now clear that in non-dividing cells one of the predominant sites where the kinase is localized is the primary cilium. The connection between NEK8 and cilia was made by the group of Lynne Quarmby, in a series of articles co-authored with Moe Mahjoub. Beginning with the observation that mutations in the *Chlamydomonas* gene *FA2* affected cell cycle progression and deflagellation, they went on to show that the gene product, Fa2p, is a NIMA family kinase that localizes to the proximal part of cilia [59, 60]. A second *Chlamydomonas* NIMA kinase, Cnk2p, was subsequently shown to have an axonemal localization, occupying in contrast to Fa2p most of the length of the flagella while controlling its size [61]. Additionally, around the same time, several of the many *Tetrahymena* NIMAs were reported to be ciliary, at least some of them being involved in the control of cilia disassembly [15, 62]. These observations lead to the hypothesis that mammalian members of the NIMA family could also be involved in the control of cilia physiology. As ciliopathies frequently include cystic kidneys among their clinical manifestations, and mutations in both NEK1 and NEK8 had been reported to cause renal cysts in mice [18, 38], the researchers investigated the subcellular localization of these two kinases [21]. They showed that in mouse kidney epithelial cell, NEK8 localized to the proximal region of the primary cilium (to a broader region than that observed for FA2p but more limited to that of Cnk2p, suggesting functional differences with these kinases), with its localization diffuse or undetectable during mitosis in cycling cells. Also importantly, the authors reported that downregulation of NEK8 does not affect cilia assembly. In contrast, NEK1 was localized to the centrosome/basal body during the whole cell cycle and as noted above was later shown to be involved in the regulation of ciliogenesis [21–23].

NEK8 has been subsequently confirmed to be a ciliary kinase in a variety of experimental systems, i.e. in mouse kidney cultured cells and epithelia [39, 58, 63, 64], rat renal epithelia [44], human fibroblasts [47], or using recombinant tagged protein in zebrafish [57], mouse [39, 45] and human cells [36, 40]. Incidentally, the study of the NIMA kinases in different organisms resulted in the current view that the family had diverged during evolution in order to coordinate cilia and cell cycle regulation, as it was found that the genomes of organisms with cilia (i.e. *Chlamydomonas*, but also humans) tend to encode more NIMA-family kinases than those that are not ciliated such as yeast [15, 16, 65]. This may be especially true if organisms have complex life cycles involving different types of cilia or ciliated cells and centrosomes that function as both spindle poles during mitosis and cilia basal bodies during interphase.

## NEK8 at the cilium: the inversin compartment

**Fig. 3:**
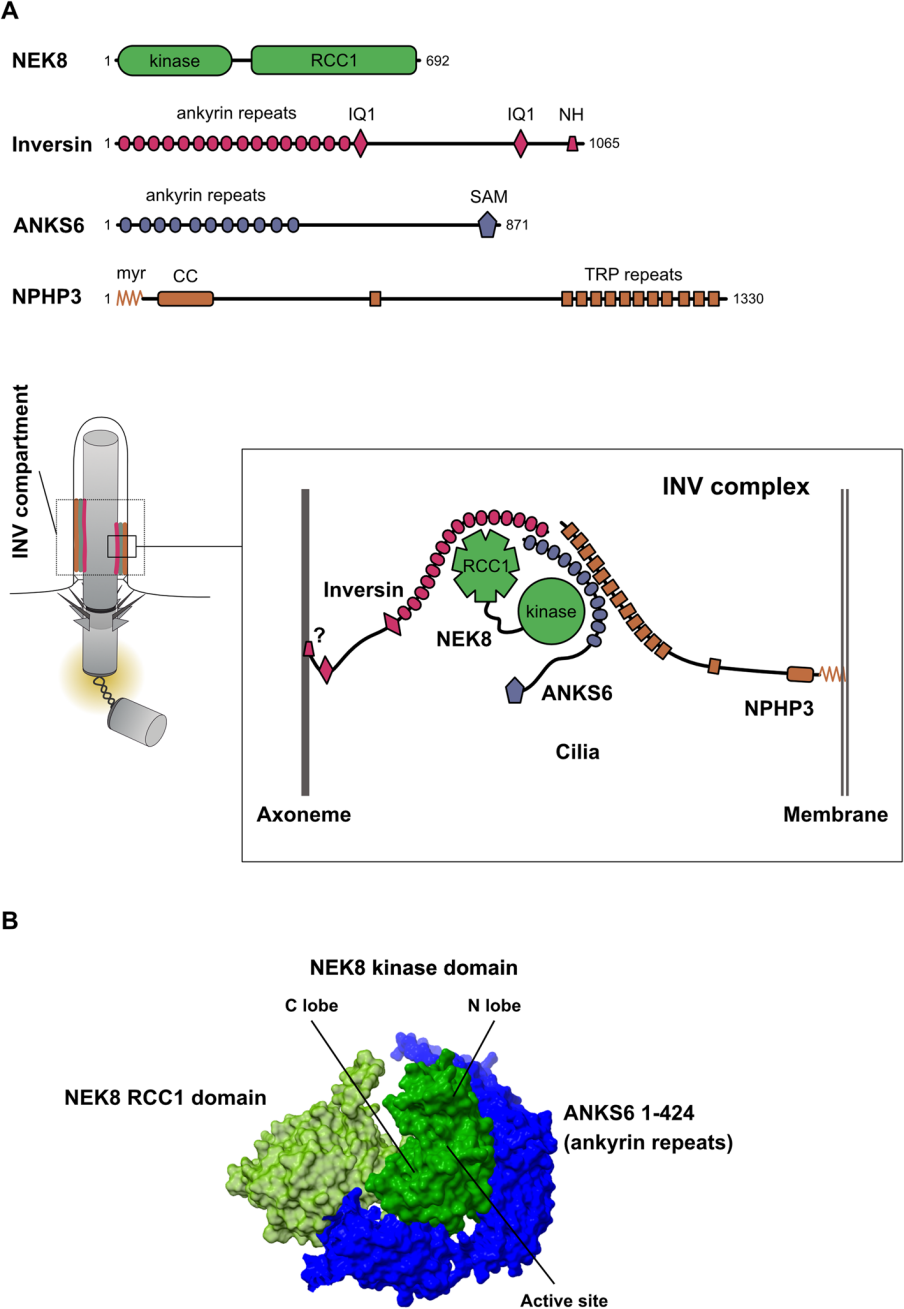
The INV complex and compartment. **A** A cartoon depicting a possible disposition of the INV complex components, taking into account published data. The proposed fibrilloid structure of the complex [40] is shown. Cilia and centrioles are not drawn to scale. *Kinase*, protein kinase domain; *RCC1*, RCC1 domain; *IQ*, IQ domain; *NH*, ninein homology region; *SAM*, sterile alpha motif domain; *myr*, myristoyl; *CC*, coiled-coil; *TRP*, tetratricopeptide. **B** The predicted interaction of NEK8 with ANKS6 ankyrin repeats. Note that ANKS6 wraps around both lobes of the NEK8 protein kinase domain, at the back of the kinase active site. Structure predicted using Alphafold [33] and modified in ChimeraX [37]

NEK8 specifically localizes to the proximal segment of the cilium [21, 64] and a breakthrough regarding the eventual understanding of its ciliary roles came after it was discovered that the kinase is part of a multiprotein complex that guides its localization to a specific ciliary region, the so-called inversin (or INV) compartment (Fig. 3A). Importantly, variants in the components of the INV complex (inversin, NPHP3 and ANKS6) cause cystic and infantile nephronophthisis, either isolated or in different syndromes that in some cases also present with laterality defects.

Inversin is an ankyrin repeat-rich protein encoded by the *INVS/NPHP2* (*Inv* in mice) gene. *Inv* or *INVS/NPHP2* variants can cause *situs inversus* and renal cysts in mice, and infantile nephronophthisis, sometimes also associated with *situs inversus*, in humans ([66–69] and references therein). Pathologies caused by variants of *INVS/NPHP2* combine features of other nephronophthisis (tubular basement membrane disruption and renal interstitial fibrosis), with those of PKD (enlarged kidneys with widespread cysts). In a similar way to what has been shown for NEK8, mutations in inversin do not seem to affect cilia structure [70]. Inversin contains a number of N-terminal ankyrin repeats followed by a C-terminal domain (notably where most of *NPHP2* mutations reside) that targets the protein to cilia, specifically between the ciliary membrane and the axoneme. The last ~ 60 residues of this domain, containing a short region with similarities to the centrosomal protein ninein, confine the protein to a distinctive segment at the base of the cilium that has come to be called inversin compartment or INV compartment. This short region can also target the protein to the centriole [71]. The molecular basis for inversin localization remains to be studied in detail. It has been suggested to be dictated by its preferential binding to a structurally defined region of the axoneme formed by microtubule doublets, that in kidney cilia comprise the proximal one third to half length of this microtubule structure [72]. This hypothesis, though, is not clearly supported by recent superresolution analysis of the INV compartment [40], as noted below. Inversin can be found associated with microtubules and precipitates with tubulin [73]. This and the presence of a region with some homology with ninein, a protein involved in microtubule anchorage at the centrosome, suggests that inversin can bind directly to microtubules, although this point remains to be confirmed. Interestingly, in cycling cells inversin localizes to centrosomes in interphase and the spindle during mitosis. The protein has different D-box destruction motifs and it interacts with components of the Anaphase-promoting complex (APC/C), resulting in cell cycle oscillations of its cellular amount [74].

The connection between NEK8 and inversin was made by the Yokoyama group [75], that reported that full length inversin localized both NEK8 and another ciliary protein called NPHP3 to the inversin compartment. In fact, NEK8 ciliary localization is completely lost in inversin-deficient cells. The data also suggested that NEK8 and NPHP3 interact with the N-terminal ankyrin repeat region of inversin and that these two proteins are dispensable for inversin localization to cilia, depending exclusively on the C-terminal region of the protein (the assembly of a normal inversin structure at the cilia, though, most probably still needs NEK8 in an undetermined manner, see [40] and below). The binding of inversin to NEK8 has been confirmed by different groups and mapped to the RCC1 domain of the kinase [41, 57, 76]. This agrees with data showing that the localization of NEK8 to the cilium is also conferred by its RCC1 domain [36]. Importantly, NEK8 has been shown in zebrafish to functionally be downstream of inversin [57].

Regarding NPHP3, a protein containing a central a tubulin-tyrosine ligase (TTL) domain and several tetratricopeptide repeats at its C-terminus, its mutations have been shown to result in cystic kidneys in mice, while loss of the protein results in *situs inversus*, heart malformation and embryonic lethality [77, 78]. In humans, variants of the *NPHP3* gene are found in a subset of infantile or adolescent nephronophthisis patients, presenting with a variety of developmental defects including situs inversus, polydactyly, retinal degeneration, heart, liver and pancreas defects and a wide range of renal system abnormalities [68, 69, 78]. As expected from [75] NPHP3 can be found in a complex with inversin [78] and NEK8 [48] in vivo. NPHP3 localization to the cilium is regulated by its myristoylation, and the protein is thought to connect the INV complex to the membrane of the cilium [79–81].

To NEK8, inversin and NPHP3, an additional functionally related partner called ANKS6 was added in 2013 [82] completing what can be called the INV complex. Like other INV complex members, ANKS6 (ankyrin repeat and SAM domain-containing protein 6, also known as NPHP16 and SamCystin) was originally identified as the product of a gene that when mutated resulted in polycystic kidneys in rats [83]. In humans *ANKS6/NPHP16* variants cause infantile onset cystic kidney disease or juvenile nephronophthisis, pathologies that are sometimes associated with enlarged kidneys and a range of developmental defects, including cardiovascular abnormalities, liver fibrosis and *situs inversus* [82]. Like inversin, ANKS6 contains a number of N-terminal ankyrin repeats, and together with this protein, NEK8 and NPHP3 it localizes to the proximal region of the cilium. ANKS6 also contains a SAM motif in its C-terminal region, that probably is like the ankyrin repeat domain involved in mediating protein–protein interactions.

The ankyrin repeat domain of ANKS6 interacts with the N-terminal half of NEK8, containing the kinase domain (see Fig. 3B). The two proteins form a robust complex that is insensitive to conditions that disassemble the rest of the INV complex and can be present in the cytoplasm before being imported into the cilium [41, 81]. The ankyrin repeat domain of ANKS6 can also bind NPHP3, being thus instrumental in bringing the INV complex together. While the interaction with inversin localizes NEK8 to the proximal cilia, the regulation of ANKS6 ciliary localization does not seem to be so straightforward, as it depends on both inversin (for its localization) and NEK8 (for its amount) [40, 41, 82]. In fact ANKS6 does more than structurally connect NEK8 to the INV complex, as it is both an activator and substrate of NEK8 protein kinase activity [41]. ANKS6 phosphorylation by NEK8 may be promoting its binding to inversin and thus regulate in part ANKS6 localization to the INV compartment. Adding an additional layer of complexity, phosphorylation of ANKS6 by NEK8 seems to be regulated by NPHP3 [81].

The most systematic study of the assembly hierarchy of the INV compartment has been done by the Jackson group using genetically engineered RPE1 cells [40]. In a remarkable article that also addresses the ultrastructure of the INV compartment, the authors show that as expected from previous data the elimination of inversin results in the loss of the specific localization of NEK8, ANKS6 and NPHP3. While the first two are lost from the cilium, NPHP3 is still ciliary, although now with a diffuse localization, suggesting that the role of inversin and its partners is to localize NPHP3 at the INV compartment but that myristoylated NPHP3 can go to the cilium independently [79, 80]. Inversin is thus the keystone of the compartment that is named after it, and recombinant forms of the protein can localize to cilia even without the other INV partners being present. But under physiological conditions endogenous inversin depends on NEK8 and ANKS6 for its correct localization, suggesting that building of the INV compartment is a complex process that may depend among other things on NEK8 phosphorylation (though NEK8 protein kinase activity does not seem to be strictly necessary for the assembly of the INV complex). Knockout of ANKS6 strongly affects NEK8 localization and the other way around, once more highlighting the interdependence of the two proteins and further suggesting that they function as a subcomplex [81]. The lack of either NEK8 or ANKS6 affects NPHP3 localization, but the elimination of NPHP3 does not affect the localization of the other INV compartment proteins, indicating that this protein is the outer element of the complex.

Using superresolution microscopy the INV compartment components are found parallel to the axoneme and separated from the transition zone, the proximal region of the cilium that controls the entry of exit of proteins to the organelle. The compartment that they define has a variable length (~ 2 μm or ~ 50% of the cilium length in RPE1 cells) that does not seem to correlate with any other known cellular structure. Inversin, NEK8, ANKS6 and NPHP3 colocalize around the axoneme, sometimes symmetrically but often accumulating asymmetrically in just one side of a subregion of the cilium in what has been called by the Jackson group “fibrilloids”, to reflect the uncertainty of whether they indeed are continuous polymers or fibrils (see Fig. 3A). These structures vary not only in length but also in number, ranging from 1 to 5 in each cilium, with an average of 3. In the model proposed by these authors, the length and number of fibrilloids and how dense they pack will determine the size, shape (i.e. symmetry) and protein density of the INV compartment. This does not seem to vary once assembled, at least regarding length [84], although photobleaching experiments indicate that there is some dynamicity to the structure [71]. The molecular basis of fibrilloid formation is not understood as none of the INV compartment components are known to oligomerize, and thus be amenable to form a scaffold around which the rest could assemble. Some of the complex components, such as NEK8 [42] and INV [85], may be able to form dimers or oligomers, though, but this needs to be studied further. It is conceivable that compartment formation could be regulated and that different cell types could assemble a bigger or smaller compartment according to their signaling needs. In fact the formation of the INV complex has been shown to be facilitated by HIF1AN, an oxygen sensor that induces ANSK6 and inversin hydroxylation [82].

Although mutations in the different components of the INV complex result in related pathologies, and available data clearly shows that the proteins are necessary for the correct development of the kidney and other tissues as well as for left–right determination, their molecular functions remain elusive.

The INV compartment is present in a variety of cell types, including mouse ductal epithelial cells, cells in the mouse embryonic node, different renal epithelial cell lines, retinal pigment epithelial cells and fibroblasts [21, 39, 40, 43, 45, 47, 64, 71, 76, 81, 86]. This suggests that inversin and its partners have a function that is common to different cilia and cell types. The INV complex is also conserved through evolution, and a similar region containing inversin, NEK8 and ANKS6 homologues has been described in cilia in *C. elegans* (see [87, 88] and references therein). The complex is connected through inversin to other cilia and basal body signaling modules, such as those formed by NPHP1, 8 and 4 and NPH5 and 6 [76] indicating that it may participate in the crosstalk between different ciliary signaling networks.

## NEK8 localization: not only cilia?

Besides the body of cilia, several groups have reported that both endogenous and recombinant NEK8 can be observed at the centrosome of cycling cells and the basal body of ciliated non-cycling cells [36, 39, 63]. Other researchers do not seem to observe this, and in fact centrosomal localization of endogenous NEK8 does not seem to be easily observed using the most widely used anti-NEK8 antibody, obtained by the Beier group against the mouse protein [38]. Cell type, but also antibody and fixation differences, see [89], may account for this discrepancy. But altogether the available data suggests that it is plausible that a pool of NEK8 is indeed centrosomal and has regulatory functions at this organelle, similar to those of other NEKs [12]. Some of these functions may be specific to cycling non-ciliated cells and distinct to those performed by NEK8 at the cilia, and they may be conceivably shared by other INV complex partners, that in some cases have been shown to also localize to the centrosome (i.e. inversin, see above).

Additionally, a pool of NEK8 may be perinuclear or nuclear, at least in cycling cells, as a result of a yet to be characterized nuclear localization signal in the RCC1 domain [36, 42, 45]. See the section about the DNA damage response below for a discussion of possible NEK8 nuclear functions.

## NEK8 protein kinase activity and regulation

NEK8 is an enzyme, a serine/threonine protein kinase, and to understand NEK8 function as well as its physiological and clinical importance we should understand how its enzymatic activity is regulated. The initial biochemical characterization of NEK8 activity came from the group of Andrew Fry, with a long history studying NEK kinases [36]. Identification of ANKS6 as a critical activator further expanded our knowledge of NEK8 [41]. However, discrepancies between the findings of these and other researchers have hindered our ability to fully understand how the protein is regulated.

The Fry group showed that recombinant NEK8 immunoprecipitated from mammalian cells is able to phosphorylate different model substrates in vitro. In the conditions used NEK8 behaved like a typical protein kinase and mutation of residues predicted to be involved in ATP binding (Lys33), catalysis (Asp128, in the conserved HRD motif) or activation (Thr162 in the activation loop, see Fig. 2) rendered it inactive. A phosphomimetic mutant of Thr162 increased NEK8 activity, thus suggesting that the kinase is regulated through the common mechanism of activation loop phosphorylation. If in vivo this is the result of autophosphorylation or, as in the case of the related NEK9, NEK8 activation loop can be modified by another upstream kinase [90], has not been addressed so far. Interestingly, NEK8 was found to be fully active when immunoprecipitated from cells, and preincubation with ATP did not further increase its activity. This is in stark contrast to NEK9, which is inactive in (interphase) cells and once purified can be activated by incubation with physiological concentrations of ATP. We have shown that the RCC1 domain of NEK9 interacts with the kinase domain keeping it inactive. In vivo, phosphorylation of NEK9 by its upstream kinases CDK1 and PLK1, followed by trans-autophosphorylation, results in NEK9 activation. This can also happen in vitro just by autophosphorylation [35, 90]. This regulatory mechanism does not seem to be in place for NEK8, with a function (and conceivably and activation mechanism) that most probably does not rely in the action of protein kinases only active in cycling cells. In fact, the kinase domain of NEK8 seems to be as active in vivo as the full length protein containing the RCC1 domain [36]. This may be explained by the fact, mentioned above, that the protein kinase and RCC1 domains of NEK8 are connected by an unstructured and flexible linker and may not interact (see Fig. 2).

Interestingly, NEK8 can phosphorylate its own RCC1 domain in vitro, suggesting that the domain is autophosphorylated in vivo in the context of the full length kinase [36]. Again pointing to regulatory differences between NEK8 and NEK9, it is worth noting that most if not all NEK9 autophosphorylation sites lay outside of the RCC1 domain [91]. The phosphorylation of NEK8 RCC1 domain seems to be related to the regulation of the kinase localization as inactive forms of NEK8 were not observed at cilia or centrosomes by the Fry group. Accordingly, the authors proposed that autophosphorylation of the RCC1 domain exposes a region that is able to interact with cilia and centrosome anchors. This is supported by data showing that kinase-inactive forms of NEK8 fail to properly localize [36, 39], although conflicting reports exist about this point [40, 41].

Conflicting results also exist regarding the activity of different NEK8 forms. Thus although other researchers [42], also observed that recombinant NEK8 was active when immunoprecipitated from cells, they reported that the *jck* mutant (G422V) showed a greatly impaired kinase activity, in conflict to what the Fry group found [36]. In contrast, Czarnecki et al. [41] reported that recombinant NEK8 is only active if coexpressed with ANKS6, and that the *jck* mutant behaved as the wild type form of the kinase. These discrepancies may be explained by the effects on recombinant NEK8 activity (and possibly ANKS6 binding) of the different N- and C-terminal tags and assay conditions used, but clearly more work is needed to establish standardized conditions to assay the activity of NEK8 and determine how the kinase is regulated.

Importantly, we do not know which physiological stimuli modulate ciliary NEK8 activity in vivo. The only change of NEK8 activity observed in response to physiological changes was also described by the Fry group, that reported that in cycling non-transformed cell lines such as RPE-1 and NIH/3T3 serum starvation resulted in the proteasome-dependent degradation of recombinant NEK8 [36]. This was accompanied by activation of the (remaining) kinase thus suggesting that cell cycle exit and possibly ciliogenesis activates a destruction program for the enzyme not incorporated at the cilium and that NEK8 could be activated once at the organelle, although this too needs to be studied further.

Altogether, the available data suggest that NEK8 is a functional kinase which activity is not regulated directly by its RCC1 domain. This domain is most probably responsible for connecting NEK8 to other proteins such as inversin and localizing it to the cilium. How could NEK8 then be regulated? The kinase lacks a regulatory region similar to that of NEK9 C-terminus, that could oligomerize it and facilitate autophosphorylation in trans (see Fig. 1). It is thus most probable that other INV complex proteins are the elements in charge of modifying the activity of NEK8, i.e. by changing the conformation of its protein kinase domain or, in view of its dependence on activation loop phosphorylation, by facilitating its transient dimerization and autophosphorylation. ANKS6, predicted to wrap around the active domain of NEK8 (Fig. 3B), and with the ability to activate different forms of the kinase [41], is ideally positioned for this. Another possibility is that the INV complex could regulate NEK8 activation loop phosphorylation by another yet to be identified ciliary kinase. In both cases, this could explain the conflicting results obtained when studying the activity of NEK8, as its overexpression as differently tagged forms combined with different cell lysis and protein isolation procedures may disrupt critical interactions resulting in proteins or protein subcomplexes with various levels of activity.

## Possible NEK8 substrates and function

Non-ciliary roles of NEK8, i.e. in relation to the centrosome or the nucleus in cycling cells, cannot be completely discarded and may account for the observation that its overexpression results in multinucleation [38, 39]. But most plausibly the main cellular function of NEK8 is related to the primary cilia, where a significant part of the kinase localizes in differentiated cells. In this view, to speak about the function of NEK8 would be to speak about the function of the INV complex, NEK8 and ANKS6 at its core. And having in mind that NEK8 is the only INV complex protein with a known enzymatic activity, it is reasonable to assume that the main role of the complex is to localize and regulate the serine/threonine kinase activity of NEK8 and thus the modification of its substrate(s) in response to yet to be identified stimuli. Whether NEK8 physiological substrates are circumscribed to ANKS6 and this protein is the sole effector of the INV complex (as suggested by [81]) or whether other NEK8 substrates exist, remain to be established. Neither inversin or NPHP3 seem to be NEK8 substrates [41]. The polycystin PC2 was suggested to be modified downstream of NEK8 [64], although available data argue against it being a direct substrate of NEK8 [41] (see “Polycystins” below).

Which could be the functions of NEK8 and the INV complex? Two functions could be expected from a ciliary kinase. It can regulate the building, maintenance and resorption of cilia structure and/or be involved in signal transduction through it. Interference experiments as well as mutations in model systems strongly suggest that NEK8 is mostly involved in ciliary signaling. The pathological results of *NEK8* variants in human patients argue in the same direction and will be treated in the following section.

Thus, several authors have convincingly shown in different systems that NEK8, like other INV compartment proteins, is not necessary for ciliogenesis (e.g. [21, 39, 40, 43, 45]. Conflicting reports, however, exist regarding the effect of NEK8 mutations on cilia length [39, 44, 47, 51, 63], suggesting that, although not crucial for ciliogenesis or the maintenance of gross cilia structure, at least in some cell types the kinase may be directly or indirectly involved in the regulation of some aspect of cilia structure and specifically its length, as other NIMA-family kinases do in *Chlamydomonas* and *Tetrahymena*.

Regarding cilia signaling, the *jck* phenotype was crucial to frame our understanding of the roles of NEK8 in it. It established that NEK8 has important functions in duct epithelial cells and that it is involved in the regulation of epithelia structure and normal kidney development. Expanding this knowledge, other mouse lines with an impaired NEK8 activity have shown that the kinase has a role during the establishment of left–right asymmetry, something that was already suggested by studies of the different INV complex proteins and of NEK8 in zebrafish. In fact NEK8 is expressed in the nodal cilia, suggesting that it may be involved, together with other INV compartment proteins, in the sensing of nodal flow during the establishment of left–right asymmetry in early development [43]. *NEK8*^−/−^ mice, besides kidney cysts, show randomized left–right asymmetry and cardiac abnormalities and die shortly after birth [43]. Similarly, homozygous *Roc* animals, containing a recessive mutation in the NEK8 kinase domain that inactivates it (Ile124Thr), show cystic kidneys but also strong laterality defects (both heterotaxy and *situs inversus*) and congenital heart disease [41]. Of note, a similar phenotype is also observed as a result of the *Streaker* mutation in *ANKS6*, that interferes with the ability of the protein to bind and activate NEK8 [41], and in a number of human patients with NEK8 variants (see “NEK8-associated pathologies in humans” below).

It is not clear why *jck* mice do not show laterality defects. One possible explanation is that the *jck* mutation (Gly448Val) may only partially interfere with the ability of NEK8 to signal, allowing enough kinase activity to be elicited in the context of the INV complex and supporting quasi-normal signalling during left–right asymmetry determination and possibly other early developmental processes. Organs like the kidney would be more severely affected maybe as a result of a higher requirement of NEK8 activity later in development. Alternatively, NEK8 Gly448Val may be a gain of function mutant, possibly as a result of its mislocalization. This seems to be suggested by the observation that *jck/Nek8*^−^ compound heterozygotes develop less severe PKD than *jck/jck* mutants [43].

Importantly, the *Roc* mutation disrupts the protein kinase activity of NEK8 [41] without affecting the assembly of the INV complex [40]. The observation that the resulting phenotype is very similar to those ensuing from loss of function mutations in different INV complex proteins strongly suggest that the whole functionally of the complex orbits around the kinase activity of NEK8. Its main function, if not the only one, may be to properly localize and control this activity during left–right determination and tissue development.

As mentioned, no conclusive data exists regarding the physiological signals that recruit INV/NEK8 signaling during organ development. Which other signaling components, if any, are involved in the transduction of these signals is also not known, although several proposals have been put forward relating INV compartment proteins with known cilia signaling pathways, i.e. Wnt [92] and Akt [85] in the case of inversin. We outline here these involving NEK8.

### Polycystins

Polycystin-1 (PC1, PKD1) and polycystin-2 (PC2, PKD2) are transient receptor potential (TRP) channel membrane proteins that function at the cilium. Polycystins function alone (PC2) or as a complex (PC2 + PC1) forming a Ca^2+^ transporting channel and they may be activated in response to mechanical stimuli (the role of cilia as calcium-responsive mechanosensors is controversial; for a recent revision of the roles of PC1, PC2 and other cilia signaling proteins, see [2]). *PKD1* and *PKD2* variants cause the most prevalent form of PKD, autosomal dominant PKD (ADPKD, which is also the most frequent renal ciliopathy). *PKD2* variants also cause laterality defects, suggesting that at least this polycystin may be functionally related to NEK8 and its INV partners. In fact, one could argue that NEK8 is ideally placed for sensing changes in cilia shape, being in the middle of a complex that spans from the ciliary axoneme to the membrane. Moreover, *Nek8*^−/−^ cells have a reduced Ca^2+^ influx in response to fluid flow, resembling PC2-defficient cells, suggesting that PC2 function may be perturbed in the absence of NEK8 [43]. PC2 expression and localization does not seem to depend on NEK8 [43], but conflicting reports exist about the relationship between the expression of NEK8 and PC1 and PC2 [48, 51, 58, 63, 64]. Importantly, it has been reported that NEK8 can interact with PC2 (but not PC1) and possibly induce its phosphorylation [64], although Czarnecki et al. suggest that PC2 is not a direct NEK8 substrate [41]. Moreover, mice model heterozygous for both *Nek8* and *Pkd1* mutant alleles show an increased frequency of cystogenesis compared with the individual heterozygous animals [58]. Thus, a functional relationship may exist between NEK8 and polycystin signaling, and the kinase may be directly or indirectly regulating the localization of the membrane proteins, their ciliary trafficking or their stability. Further studies should clarify this.

### WNT/β-catenin

WNT glycoproteins act through different complex pathways in order to regulate cell fate, proliferation, differentiation, and migration during embryonic development and tissue homeostasis. Wnt signaling, and specifically the balance between β-catenin-dependent (canonical) and non-canonical Wnt signaling, is known to be modulated by the cilium [93, 94]; moreover, inversin and possibly other *NPHP* gene products have been shown to be implicated in it through their ability to antagonize canonical Wnt signaling [95]. Regarding NEK8, the kinase has been found to interact with β-catenin promoting its degradation. Possibly in relation to this, knockdown of the kinase in cancer cell lines has been shown to decrease proliferation and its overexpression seems to correlate with shorter survival times in breast cancer patients [96]. In fact, as other NEKs (and in fact most protein kinases), NEK8 levels have been associated with cancer progression (reviewed in [97]). The relevance of these observations, their molecular basis and whether they are related to NEK8 roles at the cilium and/or in response to DNA damage (see the section below) remain to be established.

### Hippo signaling

The Hippo signaling pathway controls organ size through the regulation of cell proliferation and death. It does so through the phosphorylation of the transcriptional regulators YAP and TAZ [98]. It has been reported that NEK8 binds to TAZ and is able to promote its nuclear transport and activation (puzzlingly in a kinase-independent manner). This would possibly modulate the proliferation plan excited by the mammalian Hippo signaling pathway [46]. Note that according to this report a pool of NEK8 can be localized to the nucleus, something also suggested by [36]. Nuclear localization of NEK8 would be somehow favoured by NPHP4, a component of the ciliary transition zone, with known variants that cause nephronophthisis. A relationship between the Hippo effectors YAP and TAZ and either NEK8 or its activator ANKS6 has been observed by different groups, suggesting that this could explain some of the morphogenic defects observed as a result of pathogenic variants of these proteins [47, 48, 99]. The Hippo pathway plays a prominent role during development and has been shown to negatively regulate cilia formation, with some of its components localizing to the basal body ([100] and references therein). Whether some of the pathological manifestations of NEK8 mutations result from its interaction with this pathway remains to be determined.

### DNA damage response signaling

In 2013, somehow unexpectedly, Cimprich and colleagues proposed that NEK8 is involved in the DNA damage response (DDR), possibly connecting cilia signalling to that elicited by double strand breaks in the DNA [42, 101]. The authors showed that NEK8 downregulation in HeLa cells or its knockdown in MEFs resulted in the accumulation of DNA damage and a defective response to replication stress. Compatible results were later reported by [47], observing that NEK8 missense variants were associated to DNA accumulation in patient fibroblasts. Moreover, a role for NEK8 in the DDR is supported by the results of [102], also reporting the need of NEK8 for an efficient DDR in response to replication inhibition.

The new function of NEK8 was suggested to be the result of its ability to interact with ATR pathway components. Furthermore, it was shown that NEK8 could interact with cyclin A-CDK2 complexes regulating their protein levels and activity and suppressing them in DNA-damaged cells, that inhibit CDK complexes to regulate the firing of DNA replication origins during the S phase of the cell cycle. The authors proposed that NEK8 travels with the DNA replication fork in cycling cells, and that it is involved in ensuring normal replication fork progression. Low NEK8 levels would impair stalled fork processing, especially during the response to drugs like aphidicolin, resulting in DNA damage. Indeed, kidney cells from *jck* homozygous mice accumulated DNA damage, although whether this contributes to the observed renal *jck* phenotype remains to be established.

Puzzlingly in view of other published data, the authors also reported that, similarly to DNA damage, NEK8 downregulation reduced the frequency of ciliated cells and disrupted epithelial organization in 3D renal cell cultures [42]. Inhibition of CDK reversed the ciliary defect, linking these observations to previous reports showing that CDK inhibitors could arrest PKD progression [103].

A connection between the cilium signaling apparatus and the DDR has been suggested by other authors, and in fact other NPHP proteins (some also with ciliary functions such as CEP164/NPHP15 and NPHP10) have been involved in the response to damage and in the control of DNA repair [104]. But a number of questions remain regarding the possible implication of NEK8 in the DDR. Key results supporting an involvement of NEK8 in the signaling elicited by replication stress are observed by [42] in non-ciliated cycling HeLa cells. Ciliated cells are not cycling and it is thus difficult to see how a kinase that is active at the cilium can be affected by DNA replication stress. Moreover, the response to DNA damage involves nuclear proteins. Altogether this suggests that if NEK8 is involved in the DDR it should be through the action of a non-ciliary pool of the kinase. One can hypothesize that this could be a function performed by the nuclear pool of NEK8 that some groups have suggested that exists [36, 42]. This remains to be clarified, together with the effects that DNA damage may have on the activity of nuclear or non-nuclear NEK8 (although the activity of the kinase is shown to be required for its genome maintenance functions Choi et al. did not detect NEK8 activation in response to DNA damage [42]). Which are the relevant substrates of NEK8 in the DDR, and whether the regulation of NEK8 in this context implies some of its INV compartment partners, specially ANKS6, also remains to be established.

## NEK8-associated pathologies in humans

In 2008 the group of Friedhelm Hildebrandt identified the first pathogenic variants of *NEK8* in humans [45]. They were associated with nephronophthisis and specifically a new infantile subtype of the pathology. As the ninth gene to be associated with NPHP, *NEK8* was given the alternative name *NPHP9*. Subsequently, the work of different groups has shown that variants in *NEK8/NPHP9* and its associated INV complex genes *INVS/NPHP2* [66], *NPHP3* [77] and *ANKS6/NPHP16* [82], lead to related types of nephronophthisis or nephronophthisis-related ciliopathies (NPHP-RC; reviewed in [8]).

Specifically, patients with pathogenic variants of the *NEK8/NPHP9* gene that survive birth course with infantile nephronophthisis, cystic-dysplastic livers and hepatic fibrosis and congenital heart defects (OMIM #613824, i.e. [45]). These are probably the result of relatively subtle affectations of normal NEK8 function. A graver disruption of NEK8 (i.e. as a result of homozygous loss of function *NEK8* variants) leads to more severe phenotypes and the loss of fetal viability and has been catalogued as renal-hepatic-pancreatic dysplasia 2 (RHPD2, OMIM #615415) for its similarity to Ivemark syndrome (RHPD1). These are embryonic phenotypes with cystic/dysplastic changes in the kidney, liver, and pancreas, congenital heart disease, heterotaxy or *situs inversus* and in some cases other developmental abnormalities involving the lung, uterus, brain (i.e. cerebellar vermis aplasia) and extremities. [47–50]. They resemble the pathologies resulting from NEK8 loss of function phenotypes in mice [41, 43].

Both NPHP9 and RHPD2 are recessive pathologies, but recently an autosomal dominant PKD form resulting from variants in the region coding for the protein kinase domain of NEK8 has been described. Labelled polycystic kidney disease 8 (OMIM # 620903), it presents with enlarged cystic kidneys and is most frequently not associated with any extra-renal features [51–53]. Being the eight APKD-associated gene, *NEK8* has also been named as *PKD8*. As noted above, NEK8 may be functionally related to polycystins [64], something that could explain the link between PKD and NPHP [105] and suggest that the *NEK8* gene might be a PKD modifying gene. Note that, in mice, the *jck* mutation results in kidneys that share many characteristics with those of autosomal dominant PKD [38, 63, 105]. For a list of published NEK8 pathogenic variants see Table 1 and references therein; see also Fig. 2.

At the molecular level pathological variants of NEK8 interfere with the function of the kinase in different manners. Some variants of the gene simply result in a truncated and/or unstable protein or no protein at all, i.e. because they create a premature stop codon as a result of a nonsense or frameshift mutation, strongly affect protein stability or disrupt splicing of the mRNA. When homozygous or compound heterozygous with other severe variants, these result in RHPD2-like syndromes, i.e. laterality defects and fetal death (e.g. [47, 48, 50]), once more suggesting that there is a minimal level of NEK8 activity needed for the establishment of laterality and the normal development of different tissues and body parts (see above).

Pathological missense variants occur in both the kinase and RCC1 domains and can have a variety of effects on the protein. In the kinase domain they may disrupt kinase activity, i.e. by affecting a residue necessary to bind ATP or key to the phosphate transfer reaction. Interestingly a number of these variants are dominant, resulting in relatively mild renal manifestations [51–53]. At least one of the variants (Arg45Trp, found recurrently de novo and in individuals that in some cases present mosaicism, see Table 1) has been suggested to interfere with the kinase activity of NEK8. The observed dominant effect of this and other variants could be explained by a hypothetical dependence on trans-autophosphorylation for NEK8 activation, as an inactive NEK8 monomer could interfere with the activation of its wild type partner in the context of the dimer or oligomer. Indeed, dimerization of human NEK8 has been suggested by [42]. In addition, in *C.elegans* the NEK8 homologue (NEKL-2) has been shown to be functionally a dimer and its dimerization to recapitulate the phenotype of a constitutively active INV complex [88]. An alternative explanation for a dominant negative effect of NEK8 variants could be the binding and sequestering of a key (possibly not very abundant) substrate by the variant form of NEK8, precluding its modification by the wild type kinase.

Variants in the kinase domain could also affect the binding of NEK8 to ANKS6, interfering with the regulation of the enzyme by its INV compartment partner. Missense variants in the RCC1 domain, such as the *jck* mutant in mice, could similarly affect the interaction of NEK8 with inversin and thus proper protein localization. Interestingly, one pathological NEK8 variant (Ter693LeufsTer86; [49]) produces a frameshift that results in an elongated C-terminal region. We can speculate that the extra amino acids may also affect the interaction of NEK8 with some of its partners, e.g. inversin, impairing its normal localization and/or regulation.

## Final conclusions and open questions

Years of research by many groups has firmly established NEK8 as a ciliary protein kinase. Moreover, we now know that NEK8 lies at the center of a multimeric protein complex formed by inversin, ANKS6 and NPHP3 at the proximal region of cilia, where it defines the so-called INV compartment. It is clear that this complex is not necessary for ciliogenesis or the maintenance of cilia structure, although it may contribute somehow to regulation of these processes in some cell types. Instead, the drastic phenotypes that result from an abnormal NEK8, inversin, ANKS6 or NPHP3 function in different model organisms and in human patients indicate that the INV complex is necessary for normal ciliary signaling during the establishment of left–right asymmetry and the development of different tissues, notably the kidneys but also others such as the liver and the heart. Which is the exact function of the INV complex, which are the stimuli that elicit INV/NEK8 signaling, how they modulate the activity of the protein kinase, and which are the physiologically relevant substrates of NEK8 are open questions. The identification of ANKS6 as a NEK8 interactor and regulator that is also a substrate of the kinase has been a major breakthrough towards answering some of these, but a lot more needs to be done to answer them all. This should include 1) the elucidation of the inner workings of the INV complex regarding the control of NEK8 activity, highlighting the similarities and differences between the regulatory mechanisms of NEK8 and NEK9 and their evolutive relationship, 2) the study of how the activity levels of endogenous NEK8 change in response to different stimuli and cellular conditions and whether the kinase (possibly together with ANKS6) may be released from the cilium to the cytoplasm at some point of its regulatory cycle, and 3) the identification of the substrates modified upon activation of NEK8, possibly in a consensus sequence similar to that defined by [106] (W[LMFW]XX[(S)T][MF][KR][Ø], where X is any residue and Ø indicates hydrophobic residues; note that NEK8 does not seem to show a strong preference for any of the indicated residues; future characterization of sites modified in bona fide NEK8 substrates will confirm the usefulness of the proposed consensus sequence).

It should be especially interesting to determine whether NEK8/INV signalling is involved in mechanosensing and to establish its possible relationship with the polycystins and other relevant ciliary signalling systems. The highlighted research program could also help identify possible roles of NEK8 in actively cycling non-ciliated cells, e.g. at the centrosome or the nucleus, and clarify their relationship to those at the cilia.

Importantly, future research should enhance our ability to accurately predict the pathological consequences of novel NEK8 variants found in human patients. This knowledge should empower physicians to make more informed clinical decisions, paving the way for novel or improved personalized treatment strategies.

## Data Availability

No datasets were generated or analysed during the current study.

## Abbreviations

DDR: DNA damage response
INV: Inversin
NPHP: Nephronophthisis
PKD: Polycystic kidney disease
RHPD2: Renal-hepatic-pancreatic dysplasia 2
